# Congenital Nasolacrimal Duct Obstruction (CNLDO): A Review

**DOI:** 10.3390/diseases6040096

**Published:** 2018-10-22

**Authors:** Aldo Vagge, Lorenzo Ferro Desideri, Paolo Nucci, Massimiliano Serafino, Giuseppe Giannaccare, Andrea Lembo, Carlo Enrico Traverso

**Affiliations:** 1 Eye Clinic of Genoa, Department of Neuroscience, Rehabilitation, Ophthalmology, Genetics, Maternal and Child Health (DiNOGMI), University of Genova, 16132 Genova, Italy; mc8620@mclink.it; 2 IRCCS Ospedale Policlinico San Martino, 16132 Genova, Italy; 3 School of Medicine and Pharmacy, Department of Neurosciences, Rehabilitation, Ophthalmology, Genetics, Maternal and Child Health (DiNOGMI), University of Genoa, 16132 Genoa, Italy; lorenzoferrodes@gmail.com; 4 University Eye Clinic San Giuseppe Hospital, University of Milan, 20162 Milano, Italy; paolo.nucci@unimi.it (P.N.); massimiliano.serafino@multimedica.it (M.S.); andrealembo1984@hotmail.com (A.L.); 5 Ophthalmology Unit, Department of Experimental Diagnostic and Specialty Medicine (DIMES), University of Bologna, S. Orsola-Malpighi Teaching Hospital, 40138 Bologna, Italy; giuseppe.giannaccare@gmail.com

**Keywords:** congenital nasolacrimal duct obstruction, lacrimal apparatus, tears

## Abstract

Congenital nasolacrimal duct obstruction (CNLDO) is a common condition causing excessive tearing or mucoid discharge from the eyes, due to blockage of the nasolacrimal duct system. Nasolacrimal duct obstruction affects as many as 20% children aged <1 year worldwide and is often resolved without surgery. Available treatment options are conservative therapy, including observation, lacrimal sac massage and antibiotics, and invasive therapy. Observation, combined with conservative options, seems to be the best option in infants aged <1 year. Meanwhile, in children aged >1 year, nasolacrimal probing successfully addresses most obstructions. However, the most favorable timing for probing remains controversial. To alleviate persistent epiphora and mucous drainage that is refractory to probing, repeat probing, silicone tube intubation, balloon catheter dilation or dacryocystorhinostomy can be considered as available treatment options. Our review aims to provide an update to CNDO management protocols.

## 1. Introduction

Congenital nasolacrimal duct obstruction (CNLDO) is a common disorder in the pediatric population, causing failure in the nasolacrimal duct drainage system and presenting clinically in the overflow of tears, also called “epiphora” [1]. 

Epidemiological studies report that the prevalence of CNLDO ranges from 5% to 20% in the early phase of childhood [2,3]. MacEwen et al. found that in a cohort of 4792 infants in Britain, the prevalence of epiphora was approximately 20% in the first year of life, and almost 95% of this population showed symptoms at one month of age [3]. The pathogenesis of CNLDO lies in a mechanical obstruction located distally in the nasolacrimal duct (NLD) at the valve of Hasner, where this structure enters the nose [4]. Furthermore, most of the evidence would show the main causes of obstruction as either a pathological persistence of the membrane at the distal portion of the NLD, some bone abnormalities, or a stenosis of the inferior meatus leading to a narrowing in the lacrimal drainage system [5,6]. Moreover, the higher prevalence of CNLDO reported in premature infants compared with ones at full-term suggests the importance of the physiological development of the nasolacrimal drainage system during intrauterine life, in order to ensure the patency of the NLD [7].

The clinical presentation of the disease is mostly characterized by excessive tearing and ocular mattering. Distal obstruction at the Hasner valve is more likely to cause a mucopurulent discharge, whereas, when obstruction is near the nasolacrimal sac, (valve of Rosenmueller), it is more frequently related to a watery discharge [8,9]. While usually unilateral, CNLDO occurs bilaterally in 20% of cases [8]. The diagnosis of the disorder is confirmed by the fluorescein dye disappearance test, which evaluates the clearance of the dye from the tear meniscus in both eyes over a 5-minute period [10]. However, other causes of epiphora in infants, such as infantile glaucoma and foreign body and corneal infections should be carefully ruled out [11]. While bacteria overgrowth can occur in patients with CNLDO, it is usually delimited in the NLD and only occasionally causes conjunctivitis [1,12]. 

More recently, a higher prevalence of anisometropic amblyopia has been demonstrated in children with CLNDO (10–12%); thus in these patients, a complete eye examination, including cycloplegic refraction, should be performed with a follow-up period of at least 3–4 years [13,14]. Several studies have shown that CLNDO tends to naturally and spontaneously resolve itself within the first year of life in most cases [15,16,17,18,19]; however, in some cases, this disorder may persist beyond the first year of life, and for this reason, more practical guidelines for the management of CLNDO are needed. In fact, many ophthalmologists agree with a conservative approach as a first-line strategy, whereas a remarkable counterpart debate the primary importance of an early probing, the most common invasive approach treating CLNDO [9,20]. Thus, the aim of this study is to describe two different schools of thought in the management of this disorder and to better understand the role of medical and the surgical treatments, with a focus on the modality and timing of interventions.

## 2. Medical Treatment

The conservative approach consists of simple observation and massage of the lacrimal sac, and the application of topical antibiotics when a bacterial superinfection occurs.

### 2.1. Observation

Several studies have reported a high rate of spontaneous resolution of CLNDO within the first year of age, ranging from 32% to 95% by the age of 13 months [4,16,17,18,19]. In 1985, Paul showed a prevalence of spontaneous resolution in 15% of patients at three months, 45% at six months, 71% at nine months and 93% at 12 months in a non-randomized prospective study [19]. However, if the prevalence of resolution increases with time, most of the evidence describes higher spontaneous resolution rates in the first months of life: 80–90% in the first trimester, 68–75% in the second and ultimately 36–57% in the third [3,17,19]. Nelson et al. described a CLNDO resolution rate of 93% with conservative management in children aged 8 months or less [15]. Similarly, Noda et al. reported the patency of NLD in the population of Japanese infants treated with a non-invasive approach within 9 months of age [19]. Nonetheless, resolution of the disorder may occur even beyond the first year of life [16,21]; in this regard Young et al. found that in a multicenter, randomized clinical trial (RCT), the obstruction resolved spontaneously between the first and second year of life in 44% of the children with CLNDO [22].

In addition, bilateral CLNDO, reported in a non-negligible percentage of children (14–33.8%), is likely to reach a spontaneous resolution within the 3 months that the contralateral eye has recovered [23].

### 2.2. Massage of Lacrimal Sac

The massage of the lacrimal sac is a widely adopted conservative treatment modality, with the aim of improving the chances of resolution provided by only observation. This maneuver was first introduced in 1923 by Crigler, who elaborated a technique using a downward rotation of the thumb over the tear sac in order to break the membranous obstruction by increasing the hydrostatic pressure [24]. Subsequently, in a randomized prospective trial, Kushner showed the efficacy of this maneuver as compared to a simple massage or no massage at all [25]. Though some studies have questioned the clinical effect of this maneuver on the resolution of CLNDO [26,27], a more recent study published by Stolovitch et al. demonstrated the clinical efficacy of the Crigler maneuver in a group of 742 children with CLNDO. The results showed a success rate of 56% after the first attempt in children younger than 2 months, 46% in children aged 2 to 6, and 28% in children older than 6 months. Thus, given its efficacy, safety, and nonetheless good compliance for the patients, the authors recommended the Crigler maneuver as a first-line conservative approach for CNLDO, even in children older than 6 months [28]. In support of this evidence, the Pediatric Eye Disease Investigative Group (PEDIG) studied the cost-effectiveness of immediate office-based NLD probing in comparison with a 6-month period of conservative management followed by deferred facility-probing in a sample size of 163 children with CNLDO aged 6 to 10 months. This observational RCT revealed that in almost two-thirds of the children, the obstruction was resolved in 6 months with non-surgical management. Despite the encouraging results of a more conservative approach, the authors found better cost-effectiveness in immediate probing and, nonetheless, said that they would not neglect the possibility of office-based probing without the risks of a general anesthesia performed in the deferred intervention [29].

Furthermore, a more recent study found a statistical difference in the resolution rate of CNLDO in infants effectively treated with regular lacrimal sac massage in comparison with observed infants and infants that did not have frequent lacrimal sac massages (96.2% vs. 77.7%, *p* = 0.001) [30]. These results suggest the importance of the Crigler maneuver, the role of which should be emphasized to parents because of the high success rate and the additional benefit afforded by the “wait and see” approach.

Ultimately, given the optimal CNLDO resolution rate, the good compliance in patients and the consistent possibility of avoiding more invasive intervention such as probing, simple observation of the infant in addition to the correct execution of the lacrimal sac massage should be considered as the first-line treatment in the management of CNLDO in the first 12 months of life. 

### 2.3. The Role of Antibiotics

Several studies have focused their attention on the use of antibiotic drops in combination with conservative therapy for CNLDO [17,31,32]. However, there is no evidence of a significant clinical effect of antibiotic eye drops in the resolution of the disorder [32,33]. Moreover, it has been demonstrated that patients with CNLDO would not show differences in the bacterial flora composition of the conjunctiva in comparison with normal subjects [34]. Thus, the administration of antibiotic drops should balance the possibility of avoiding the local spread of an infectious process from the lacrimal sac with the risk of developing resistant flora causing a NLD chronic infection. In fact, the irrational use of antibiotic drops, especially in premature infants, not only would not promote CNLDO resolution, but also would facilitate the overgrowth of resistant bacteria in the nasolacrimal system given the higher susceptibility of these individuals [34,35].

On the other hand, the administration of topical antibiotic therapy has its rationale only when symptoms of discharge are present [1,35]. Moreover, an infection of the nasolacrimal drainage system may lead to severe complications such as orbital and preseptal cellulitis, in which urgent hospital admission with intravenous antibiotic treatment is required [20].

In conclusion, most of the studies agree that antibiotic therapy is indicated only with the clinical evidence of infection, but not in the conservative management of CLNDO.

### 2.4. Invasive Treatment

The first-line invasive treatment in the management of CLNDO consists of irrigation in conjunction with probing. Other second-line strategies adopted in case of probing failure are repeated probing, silicone tube intubation and balloon dilatation of the lacrimal drainage system.

### 2.5. High-Pressure Irrigation

High-pressure irrigation, as the lacrimal sac massage, creates positive pressure in the nasolacrimal system in order to resolve the obstruction and therefore should be considered as an intermediate procedure between the conservative and the invasive management of CNLDO [36]. Several studies reported that the overall success rate ranges from 33 to 100% [29,35]. In this regard, Alagoz et al. showed a CNLDO resolution rate of 81.8% with high-pressure irrigation at the first attempt in children aged 7 to 12 months and 76.5% in children aged 13 to 17 months. Moreover, although this treatment modality is less invasive than probing, damage of the lacrimal canaliculus can occur during the procedure with the canula [37]. In addition, a retrospective study by Isaza et al. revealed that probing without irrigation was overall successful in 83.5% of children with CNLDO (respectively 90.2% in children younger than 2 years and 78.9% in children older than 2 years), similarly the results shown by traditional probing combined with this procedure [38]. Thus, as most of the evidence suggests, high-pressure irrigation should be considered more as an ancillary procedure preceding probing, which remains the first-line invasive treatment for CNLDO [35,36].

## 3. Primary Probing

Probing of the nasolacrimal system is traditionally the most commonly adopted surgical procedure in the management of CLNDO [39,40,41,42]. This procedure, which requires passing a probe down to the distal portion of the nasolacrimal system in order to break the obstruction, may be performed either in office with only the administration of local anesthetics or in the operating room in a surgical facility with general anesthesia [9]. In recent years, two different philosophies have been debating about the optimal timing of intervention; the first one suggests an early primary probing (6–9 months) because even if it is technically more challenging to manage an awake and often crying child, it would allow for avoiding the risks of general anesthesia and, according to some authors, the potential development of fibrosis due to the prolonged chronic inflammation of the NLD [20,23,43]. However, subsequent studies found no presence of inflammatory-induced fibrosis in children with CNLDO [34]. On the contrary, the second school of thought recommends a late primary probing (beyond 12 months), since the procedure under general anesthesia requires less technical skills for inexperienced surgeons and, more importantly, it has shown success rates comparable to the early intervention [21,22,44].

Primary early probing is typically performed under topical anesthesia in children younger than 12 months; however most of the evidence suggests a resolution rate of CLNDO ranging from 75% to 89%, with comparable results in children older than 12 months [40,45,46]. Furthermore, in two non-randomized, multicenter studies, PEDIG analyzed the clinical efficacy of early probing in 304 children aged 6 to 15 months and revealed an overall success rate of 75% (95% CI, 70–80%), with lower results in bilateral CLNDO compared to unilateral CLNDO (63% vs. 80%; relative risk (RR) = 0.78) [46].

On the other hand, primary late probing has shown an optimal success rate (75–80%) comparable with early probing, also in older children with CLNDO younger than 3 years old [47,48,49,50]. In particular, several studies reported optimal results in children beyond the first year of age, with a resolution rate ranging from 76.8% to 89% in children aged 13 to 18 months, 54% to 88.6% in children aged 18 to 24 months and 33% to 71.7% in children aged 24 to 36 months [51,52,53]. In line with this evidence, Rajabi et al. reported in a non-randomized prospective, interventional study including 343 children with CNLDO, an overall primary late probing success rate of 75.8%, more specifically 85% in children aged 2 to 3 years old, 63% in children aged 3 to 4 years old, and 50% in children aged 4 to 5 years old [54]. Moreover, in a retrospective study including 246 eyes of 177 children with CLNDO aged 0 to 9.8 years, Napier et al. reported a success rate of 76% of primary priming as a first-line intervention, regardless of gender, age and complexity of obstruction [55].

The already-mentioned PEDIG randomized clinical trial comparing early office-based probing with a 6-month period, followed by late probing in a surgical facility, reported similar resolution rates between the two interventions. In this regard, the authors found that early probing reduced overall symptoms of 3 months and was slightly more cost-effective in comparison with the deferred procedure; however, the possibility of resolving CLNDO in two thirds of the patients with only simple observation should be carefully taken into consideration [29]. In fact, probing is an invasive and blind procedure, which is not free from complications such as bleeding, damage of the nasolacrimal system and of the adjacent structures and inflammation with subsequent NLD fibrosis [9]. Young et al. reported bleeding from the lacrimal punctum during the procedure in 20% of patients caused by the formation of a false passage [22]. 

More recently, endoscopy-assisted nasal probing has improved the success rate of this procedure and at the same time has reduced the likelihood of complications [56,57,58]. The endoscopic technique is performed under general anesthesia often through the multidisciplinary collaboration with other specialists like otolaryngologists, allowing for a direct visualization of the nasolacrimal system and the possibility to fracture the inferior turbinate in case of bone abnormalities [59,60]. Thus, given the invasiveness and high skills required by this intervention, endoscopic nasal probing should be considered an efficient second-line treatment for children with CLNDO.

In conclusion, probing has revealed promising results in the management of CLNDO, however an open debate about the optimal timing of the intervention still persists. The risk-benefit ratio of early probing should be thoroughly weighed, however, since the conservative treatment has shown to be safe and effective in the majority of the patients and late probing offers comparable results with the office-based procedure in children older than 12 months, acting as a convincing second-line strategy.

## 4. Repeated Probing

In the case of primary probing failure, patients usually develop excessive tearing and crusting within 6 weeks of the intervention [1]. For this reason, repeating the procedure under general anesthesia after a period of at least 1 month could be a viable option [36]. 

In this regard, PEDIG analyzed the clinical efficacy of repeated probing in children with relapsing CNLDO aged 6 to 48 months in a multicenter, non-randomized, prospective study, visiting the patients one month and six months after surgery, respectively; they found an overall success rate of 56% after the 6-month follow-up period [61], similar to the results reported by Kakowitz et al. with a secondary probing resolution rate of 52% in children aged 6–18 months and 18% in children aged 18–24 months [51]. This evidence suggests an important reduction in the success rate of secondary probing, likely due to the complications of the primary procedure, such as cicatricial stenosis or false passage formation [35,57]. 

## 5. Other Interventions

Several studies have investigated the clinical efficacy of other surgical interventions in the management of both simple and complex CLNDO. 

Nasolacrimal intubation is an invasive technique initially thought for the resolution of persistent CLNDO, consisting of the placement of a silicone tube stent in one or both canaliculi [19,62]. Most of the studies reported a resolution rate of silicon intubation treating complex CLNDO after failed probing ranging from 62% to 100% [51,63,64,65]. Lacrimal tubes are usually left in situ for a variable period from 2 to 6 months; however, a period of longer than 3 months of intubation has shown a higher success rate in older children [1]. Moreover, the improvement in skills and experience and the introduction of monocanalicular tubes has allowed clinicians to adopt this procedure in the primary management of CLNDO [23,66]; in particular, PEDIG recruited children with CLNDO aged 6 months to 45 months in a multicenter, prospective, non-randomized study, to undergo primary silicon intubation for a 2–5 month-period and found a success rate of 90% in patients not lost in terms of follow-up. However, in spite of the remarkable results, there are still some limitations to the study: firstly, no control group was reported, and secondly, the high rate of tube removal (41%) before the 2-month period indicates a high dropout rate due to the low compliance of the patients [23].

In addition, several studies reported better efficacy and compliance of monocanalicular intubation than bicanicular intubation because the former procedure allows a simpler insertion of the tube and avoids sedation during stent removal [67,68,69]. Moreover, a recent retrospective study published by Eustis et al. investigating 186 eyes with CNLDO reported a higher success rate of monocanilicular intubation in comparison with bicanalicular intubation (93.18% vs. 78.75%, *p* = 0.00653) [70]. Furthermore, in the above-mentioned study published by Napier et al., a resolution rate of 92% with intubation as a secondary intervention was shown in comparison with 67% in patients treated with repeated probing [55]. 

Although nasolacrimal intubation has shown considerable results in the management of CLNDO, the invasiveness of this procedure is by necessity associated with some complications, such as the already-mentioned significant dropout rate, damage of the puncta, corneal or conjunctival abrasions and granuloma formation, and thus, should be regarded more as an effective second-line strategy [20,23,65,71].

Balloon catheter dilatation is a more recent procedure which works by dilating the NLD through balloon inflation and was shown to reduce the probing-induced complications [72,73]. In a non-randomized, prospective, multicenter study by PEDIG enrolling 159 children aged 6 to 48 months after failed probing, balloon catheter dilation demonstrated a resolution rate of 77% (95% CI = 65% to 85%) in comparison with 88% of the intubation group (CI = 74% to 91%) over a 6-month follow-up period [74]. A recent study published by Hu et al. reported a comparable success rate displayed by both balloon cathether dilatation and nasolacrimal intubation (90.3% vs. 87.6%); however, the former exerted a stronger efficacy in the resolution of complex CLNDO in comparison with intubation (86.1% vs. 64.7%) [75].

Hence, given also the high cost of the equipment required for the procedure, balloon catheter dilatation should be considered an efficient treatment modality in the management of complex cases of CLNDO [31].

If all these multiple procedures have produced no results, or there are still complex cases of bony obstruction, dacryocystocele and dacryocystitis, the last resort for patients is the dacryocystorhinostomy surgical procedure [76,77]. In this respect, the more recent endoscopic technique has improved the success rate of the procedure and decreased postoperative complications due to the external approach, reducing the postoperative course [78,79]. See Table 1.

## 6. Conclusions

CLNDO is a relatively common disorder in the pediatric population (5–20%), mainly due to the persistence of a membrane in the distal portion of the NLD. The higher association with anisometropic amblyopia should not be neglected, and therefore, children with CLNDO should be followed up with a comprehensive eye examination for at least 3 or 4 years.

The medical management of the disorder has revealed a high success rate; in fact obstructin usually resolves with a conservative approach within the first year of age in two-thirds of children; for this purpose, parents should be properly instructed to perform a correct Crigler maneuver 2–4 times a day, in order to increase the chances of a CLNDO spontaneous resolution. Moreover, given the possibility of spontaneous resolution also beyond the first year, a rational choice could be postponing invasive treatment even after 15–18 months of age. On the contrary, there is no evidence about the efficacy of antibiotics in the resolution of CLNDO, and for this reason, they should be administered only in the case of clinical evidence of infection.

Probing is considered the first-line invasive treatment for CLNDO; more specifically, early probing in the office has shown comparable results with late probing under general anesthesia in a surgical facility, which can be performed with decent results within 3 years of age. Hence, given this evidence, it is reasonable to prolong the conservative management of CLNDO as long as possible and to shift to the invasive procedure when the former strategy has failed. 

However, both early and late probing have been described as efficient and relatively safe treatment modalities for the patients. Some ophthalmologists may adopt nasolacrimal intubation as a first-line strategy in the management of CLNDO. 

Furthermore, in the case of probing failure, balloon catheter intubation and endoscopic dacryocystorhinostomy, as a last resort, have proven to be viable and effective second-line treatment options. 

## Figures and Tables

**Table 1:** Clinical efficacy comparison between the treatment modalities in the management of congenital nasolacrimal duct obstruction (CLNDO) following the criteria set by Oxford-Centre for level of Evidence-Based medicine and grade of recommendations Assessment, Development and Evaluation (GRADE).

Treatment Modality	Pros	Cons	Levels of Evidence	Grade of Recommendations
Simple observation [15,19]	High compliance for the patients No invasiveness Good spontaneous resolution rate	Alone less effective than Crigler massage. Prolonged time less efficacy of probing	1a	B
Nasolacrimal sac massage [25,30]	High compliance, no invasiveness, Increased spontaneous resolution rate	Prolonged time less efficacy of probing	1a	A
Antibiotics [17,31]	Efficacy with symptoms of infection	No efficacy in CLNDO management	1c	D
Early probing [9,40,46]	High CLNDO success rate, No general anesthesia	Invasive procedure in children who could resolve spontaneously CLNDO	1a	A
Late probing [9,54]	High CLNDO success rate even in children older than 1 year. Possibility to wait for spontaneous CLDNO resolution	Invasive procedure under general anesthesia, experienced ophthalmologist required	1a	A
Repeat probing [61]	Simple second-line invasive strategy	Lower efficacy than primary probing	1a	C
Nasolacrimal duct intubation [66,70]	High success rate after failed probing, especially monocanalicular tube	Invasive, general anesthesia required, High dropout rate	1a	B
Balloon catheter dilatation [73,74]	High success rate after failed probing	Invasive procedure, General anesthesia High equipment cost	1a	B
Dacryocystohinostomy [78,80]	High success rate for complex CLNDO especially with endoscopic procedure	Invasive procedure General Anesthesia Postoperative complications High equipment cost	1c	B

Grade of recommendations: A = High; B = Moderate; C = low; D = Very Low; Levels of Evidence: 1a = Systematic reviews (with homogeneity) of randomized controlled trials; 1c = All or none randomized controlled trials.
